# Quantitative Measurement of Eyestrain on 3D Stereoscopic Display Considering the Eye Foveation Model and Edge Information

**DOI:** 10.3390/s140508577

**Published:** 2014-05-15

**Authors:** Hwan Heo, Won Oh Lee, Kwang Yong Shin, Kang Ryoung Park

**Affiliations:** Division of Electronics and Electrical Engineering, Dongguk University, 26 Pil-dong 3-ga, Jung-gu, Seoul 100-715, Korea; E-Mails: gjghks@dgu.edu (H.H.); b215p8@dgu.edu (W.O.L.); skyandla@dgu.edu (K.Y.S.)

**Keywords:** eyestrain, gaze estimation, edge strength, 3D stereoscopic display

## Abstract

We propose a new method for measuring the degree of eyestrain on 3D stereoscopic displays using a glasses-type of eye tracking device. Our study is novel in the following four ways: first, the circular area where a user's gaze position exists is defined based on the calculated gaze position and gaze estimation error. Within this circular area, the position where edge strength is maximized can be detected, and we determine this position as the gaze position that has a higher probability of being the correct one. Based on this gaze point, the eye foveation model is defined. Second, we quantitatively evaluate the correlation between the degree of eyestrain and the causal factors of visual fatigue, such as the degree of change of stereoscopic disparity (CSD), stereoscopic disparity (SD), frame cancellation effect (FCE), and edge component (EC) of the 3D stereoscopic display using the eye foveation model. Third, by comparing the eyestrain in conventional 3D video and experimental 3D sample video, we analyze the characteristics of eyestrain according to various factors and types of 3D video. Fourth, by comparing the eyestrain with or without the compensation of eye saccades movement in 3D video, we analyze the characteristics of eyestrain according to the types of eye movements in 3D video. Experimental results show that the degree of CSD causes more eyestrain than other factors.

## 1. Introduction

With the popularity of 3D stereoscopic displays, 3D stereoscopic content has been distributed widely through various types of media, such as 3D movies in theaters, 3D TV, and 3D mobile devices. Currently available 3D stereoscopic displays require the user to wear anaglyph, passive or active shutter glasses, although some 3D stereoscopic displays that are not based on glasses have been commercialized recently. In spite of the maturity of 3D eyeglass displays, eyestrain from viewing them is caused by various factors and remains a problem that must be overcome. To solve this problem, we require a method that can measure the degree of eyestrain accurately and objectively.

Previous research can be categorized into subjective [1] and objective methods [2–11]. Research that analyzes eyestrain using subjective methods can be influenced by daily conditions, states, and the perception of participants. In addition, these methods cannot be used for online measurement of eyestrain when a user is viewing the display, because such research requires participants to answer questionnaires. Conversely, objective methods can be used to measure eyestrain using physiological signals such as accommodation response, accommodative-convergence over accommodation ratio, eye pressure, blinking rate (BR), pupil accommodation speed, electrical activity of the heart, galvanic skin response, skin temperature, brain signal, and refraction [2–11]. Changes in blood pressure and heart activity for participants that were viewing a stereoscopic movie were measured using electrocardiography (ECG) [2]. In another research, event-related potential based on brain activity is used for measuring 3D visual fatigue [3]. Magnetoencephalography (MEG) is a brain scan imaging technique that is used to measure asthenopia by stereoscopic display [4]. In a previous research [5], autonomic nervous system responses were measured based on heart rate, galvanic skin response, and skin temperature with subjects watching 2D or 3D displays.

In previous research, eye refraction or pupil responses, including accommodation, were measured on participants viewing 3D stereoscopic displays [6–8]. Although accommodation response, refraction, blood pressure, ECG, brainwave, and MEG measurements were accurate, the study required inconvenient devices or multiple electrodes to be attached to the participant's body, which can be uncomfortable and become another factor that contributes to user fatigue.

In subsequent research in this field, researchers have attempted to measure eyestrain based on eye BR or the speed of pupil accommodation using a camera vision system [9–11]. These methods are based on the principle that, as eyestrain increases, the human eye blinks at a higher rate and the speed of pupil accommodation is slower. However, these studies do not consider the user's gaze position and gaze detection error on the display when measuring eyestrain. In other words, these studies use the average value of each factor that causes eyestrain, such as edge difference, scene change, hue variance, edge, and motion information, in the current entire image, instead of the specific area where the user is looking. Lee *et al.* applies an eye foveation model that considers the user's gaze position and gaze error in order to measure the eyestrain according to hue variance, edge, and motion information in 2D displays [11]. Although Lee *et al.* define the circle region where the user's gaze position exists by considering the gaze estimation error; they do not determine the gaze position that has a higher probability of being the correct one inside the circle region. In addition, they do not measure the eyestrain according to various factors in the 3D stereoscopic display.

In a previous research [12], Van der Linde introduced the method of foveation and focus compression scheme, and showed the efficiency of his proposed method based on the compression rates. In other research [13], Cöltekin provided an overview of space variant image coding for stereoscopic display and the findings from his research based on foveation for stereoscopic imaging. In addition, he presented the experimental results from a stereoscopic foveation implementation. In [14], Duchowski *et al.* reviewed previous techniques for gaze-contingent displays considering foveation for peripheral level-of-detail management, 3D visualization, and stereoscopic imaging. However, a specific gaze detection algorithm has not been used or provided in previous studies [12,13]. In addition, these studies do not consider the gaze detection error and the position that has a higher probability of being correct inside the circle region when defining the foveation saliency map or model.

Therefore, we propose an innovative method to analyze eyestrain on an anaglyph (red-green glasses) stereoscopic display considering an eye foveation model that is based on the viewer's gaze position and gaze error, and on the gray edge information.

Although the stereoscopic display that uses passive or active shutter glasses is more advanced, we chose the anaglyph stereoscopic display to measure eyestrain for this research because of the following advantages: first, the passive or active shutter-based method cannot be used on conventional 2D displays because the shutter requires additional equipment on the display. However, the anaglyph-based method can be used on conventional 2D displays without the use of additional equipment. Second, anaglyph glasses are inexpensive and lighter than passive or active shutter-based glasses.

By wearing an eye-movement capturing device that resembles eyeglasses and attaching four near-infrared (NIR) illuminators to the monitor's corners, we can easily measure the eye BR and gaze position of a user. Gaze estimation that considers the human field of vision (FOV) and average gaze error is used to measure more accurately and reliably a user's eyestrain at specific gaze positions within the entire 3D display area. In addition, the circular area where the user's gaze position exists is defined based on the calculated gaze position and gaze estimation error. Within this circular area, one position where edge strength is maximized can be detected, and we determine this position as the gaze position that has a higher probability of being the correct one. Based on this gaze point, the eye foveation model is defined. We measure the correlation between the degree of eyestrain and the causal factors of eyestrain, such as the degree of change of stereoscopic disparity (CSD), stereoscopic disparity (SD), frame cancellation effect (FCE), and edge component (EC) on a 3D display. By comparing the eyestrain in a conventional 3D video and an experimental 3D sample video, we analyze the characteristics of eyestrain according to various factors and the types of 3D video. In addition, by comparing the eyestrain with or without the compensation of eye saccades movement in a 3D video, we analyze the characteristics of eyestrain according to the types of eye movements in a 3D video. Our gaze tracking system does not measure gaze in the 3D space (accommodation, convergence), but measures gaze in the 2D space (X and Y position on a monitor).

This paper is organized as follows: in Section 2, we explain the proposed devices and algorithms. In Section 3, the factors of visual fatigue caused by the 3D display are shown. In Section 4, we provide the experimental setup and results. In Section 5, the discussions of the experimental results are shown. Conclusions and future work are presented in Section 6.

## 2. Proposed Device and Methods

### 2.1. Eye Capturing Device

**Figure 1.**
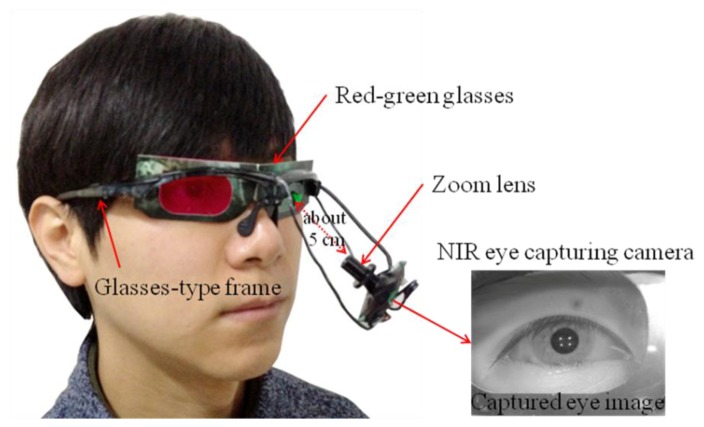
Eye capturing device and captured eye image.

Our proposed device for capturing eye-movement includes a small web-camera with a zoom lens and the interface of a universal serial bus [11]. The camera can capture images at a resolution of 640 × 480 pixels at a speed of 15 fps. It is mounted under the left eye of an eyeglass device using a flexible frame, as shown in Figure 1 [11]. The zoom lens provides a highly magnified eye image.

To acquire clear eye images and calculate the user's gaze position, four NIR illuminators, with a wavelength of 850 nm that does not dazzle the user, are attached to the four corners of the monitor [11]. To capture NIR images robust to environmental visible light, the NIR cutoff filter of the camera is eliminated, and a NIR transmission filter is included in the camera [11].

### 2.2. Gaze Tracking Method

With a captured eye image, the gaze tracking algorithm works as follows. To extract the center of the pupil in the eye image, a circular edge detection (CED) algorithm is employed. The algorithm approximates the pupil position where the difference in gray levels between two adjacent circular templates is maximized [11]. Because the pupil is not a complete circle and its shape in the eye image can be distorted by the eye capturing camera, the following steps are performed to accurately detect the pupil position [11]. Local binarization is performed in the area that is defined based on the initial pupil center obtained using the CED algorithm. Then, component labeling, size filtering, filling of specular reflection (SR) area, and calculation of the geometric center of the remaining black pixels at the pupil center are performed. Four NIR illuminators (attached to the monitor corners) produce four SRs in the eye image (as shown in Figure 1), which represent the positions of the monitor corners [11,15]. These reflections are detected in a predefined area based on the position of the pupil center, and they are detected using binarization, component labeling, and size filtering [11,15].

With the detected pupil center and the four SR positions, gaze position on a monitor is calculated using a geometric transform method [11,16]. In general, there are differences between the visual and pupillary axes called angle Kappa; this difference is compensated through user-dependent calibration (each user is initially requested to gaze at the center of the monitor) [11,15]. The average root mean square (RMS) error of gaze detection is estimated at approximately 1.12° [11,15].

### 2.3. Obtaining the Weighting Mask for the Eye Foveation Model

**Figure 2.**
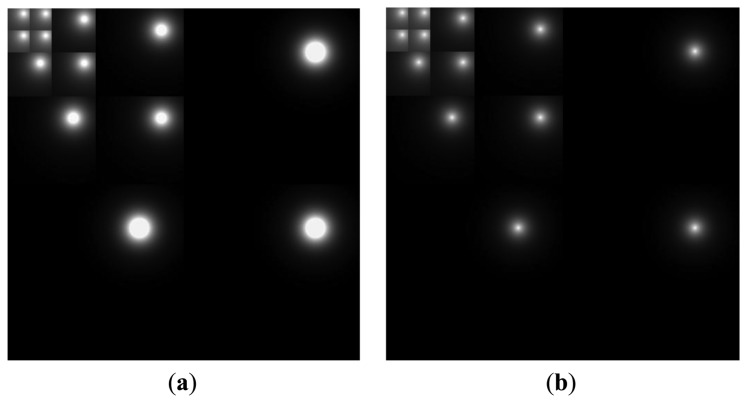
Foveation-based contrast sensitivity mask in wavelet domain: (**a**) using the circle region considering the gaze position with average gaze error of 1.12° [11]; (**b**) using the gaze position that is determined based on the maximum edge magnitude inside the circle region (proposed method).

Previous research shows that the sensitivity to contrasts in human vision decreases with an increase in the distance from the user's gaze position [12–14,17,18]. In such research, this sensitivity change can be calculated using a foveation model. The spatial resolution of the human visual system is highest at the user's gaze point (foveation point), and it decreases with an increase in the distance from the foveation position (eccentricity). In other words, humans perceive the image at a gazed position to be a high-resolution image, whereas the surrounding area, which is far from the gaze position, appears to have a comparatively lower resolution [11]. We used this foveation model to accurately calculate the factors that cause eyestrain when viewing 3D images. We measured the viewer's eyestrain according to its causal factors, such as the degree of CSD and the FCE of the 3D display (see Section 3). These factors can be quantitatively calculated from each image frame. However, we calculate these factors over the entire image that is changed according to the sensitivity model of the human FOV, similar to [11]; in other words, the foveation model of changing image resolution shown in Figure 2.

The human foveation model can be calculated based on [11,17,18], and the visual sensitivity model shows that the weighting mask of the single foveation point (gaze point) is found in a discrete wavelet domain, as shown in Figure 2 [11,18]. Based on previous research [11,19], we obtain the mask using a four-level discrete wavelet transform (DWT) that is based on the Daubechies wavelet bases. In each sub-region, the area that corresponds to higher contrast sensitivity (higher image resolution) is shown as a brighter gray level, and the area that corresponds to lower sensitivity (lower image resolution) is represented by a darker gray level [11,18]. Because the human eye is more sensitive to lower-frequency components, the LL regions (low frequency in the horizontal and vertical directions) are further decomposed using a four-level DWT, as shown in Figure 2.

Furthermore, in order to obtain a more accurate visual sensitivity foveation model, the average gaze error (1.12°) of our gaze tracking method is considered in the mask of the wavelet domain, as shown in Figure 2a [11]. However, this method has the problem that all the positions within the circle region where user's gaze position exists (the white circle in Figure 2a) are considered to have the same sensitivity, because the gaze position that has a higher probability of being the correct one is not known within the circle region. Given that a user actually gazes at one position within the circle region, we determine the gaze position, which has a higher probability of being the correct one, using edge information. In previous research [20], it was found that the edge information of high spatial frequency dominates the probability of eye gaze position. Therefore, we find the gaze position that has a higher probability of being the correct one where the magnitude of the filter response by four directional Sobel masks is maximized within the circle region.

**Figure 3.**
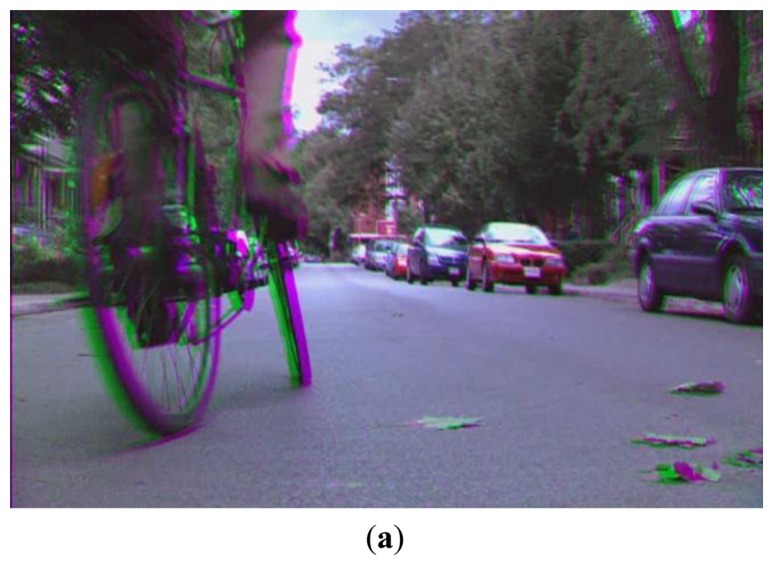

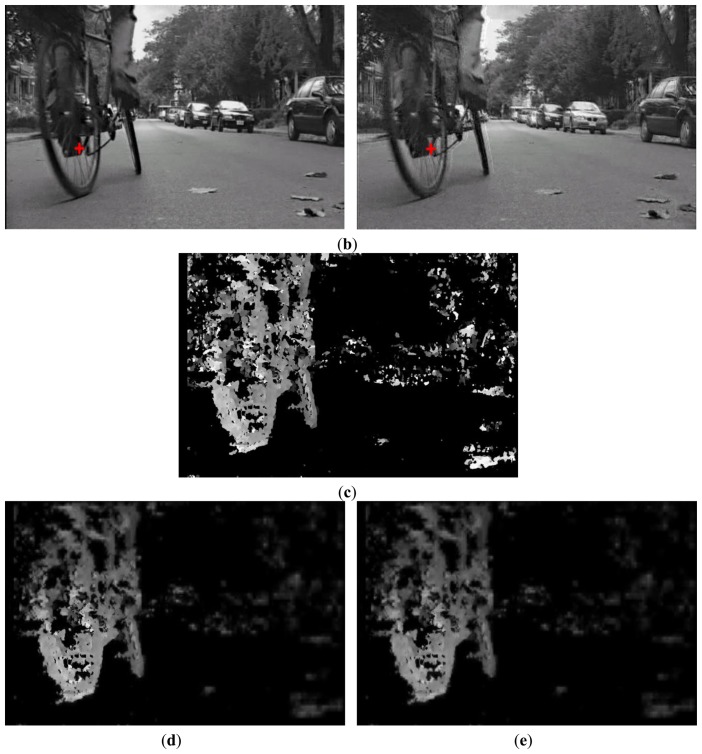
Examples of stereoscopic, left and right, disparity, and transformed images (where foveation model is applied): (**a**) original 3D stereoscopic (red-green) image; (**b**) left (green) and right (red) images of (a); including the foveation point as a red crosshair; (**c**) disparity image of (b) using the OpenCV library [21]; (**d**) transformed disparity image of (c) based on the foveation point of the red crosshair of (b) by the previous method [11]; (**e**) transformed disparity image of (c) based on the foveation point of the red crosshair of (b) by the proposed method.

The Sobel mask is applied to left and right images, respectively. The left and right images are the images separated from the original 3D stereoscopic (red-green) images of Figure 3a, and they are represented as gray images as shown in Figure 3b. Therefore, we do not use the color gradient, but the gray gradient that is obtained in both the left and right images. Because there are various edge directions in an image, we use four directions (0°, 45°, 90°, and 135°) Sobel masks.

In our research, a user wears the red-green glasses shown in Figure 1. Therefore, a red or green component is perceived by the user, instead of the entire color obtained through the RGB components. In addition, the circle region defined by gaze detection error is extremely small; consequently, the edge orientation within this region is similar to that in the surrounding area because of the characteristics of continuity of neighboring pixel values in an image. Thus, we consider only the edge information in this research based on [20], although the perceptual (visual) saliency of humans is a more complex concept that is affected by various factors, including color, intensity, orientation, and motion [22,23]. These various factors will be considered in future work.

Consequently, the original equation for the distance from the foveation point of Equation (1) [11,18] can be revised into Equations (2–4) in our research:

(1) $$d_{\lambda ,\theta }\left(x\right)=2^{\lambda }{\left\|x-xf_{\lambda ,\theta }\right\|}_{2}\text{for}\;x\in B_{\lambda ,\theta }$$

where **xf** = (*xf*_1_, *xf*_2_) is a user's gaze position and **x** = (*x*_1_, *x*_2_)^T^ (pixels) is any point in the image. In addition, λ is the wavelet decomposition level and θ shows the LL, LH, HL, and HH sub-bands of the wavelet transform. Here, LL and HH are the low and high frequency components both in the horizontal and vertical directions, respectively. LH and HL are the low frequency component in one direction and the high frequency component in the other direction among horizontal and vertical directions, respectively. **B**_λ,θ_ is the set of wavelet coefficient positions in sub-band (λ, θ) [11]:

(2) $$d_{\lambda ,\theta }\left(x\right)=2^{\lambda }{\left\|x-{\text{xcf}}_{\lambda ,\theta }\right\|}_{2}\text{for}\;x\in {\text{B}}_{\lambda ,\theta }$$

(3) $${\text{xcf}}_{\lambda ,\theta }\underset{x}{\arg\max}\left(\text{TMSM}\left(x\right)\right)$$

(4) $$\text{TMSM}\left(x\right)=\sum_{i=0}^{3}\left|{\text{MSM}}_{i}\left(x\right)\right|,\text{if}\;2^{\lambda }{\left\|x-xf_{\lambda ,\theta }\right\|}_{2}<Nv\tan e$$

where *N* is the width of an image, and *v* is the viewing distance measured in image width from the eye to the image plane [11,18]. Therefore, *Nv* is the calculated Z distance between a user's eye and the image plane. If we assume that *e* is the gaze tracking error in degrees, the radius of the circle region (where user's gaze position exists) determined by the gaze tracking error can be *Nυ* tan *e* in the image plane [11]. Consequently, the condition (if2*^λ^*‖**x − xf***_λ_*,*_θ_*‖2 < *NV* tan *e* of Equation (4)) represents the case where one point (**x**) belongs to the circle region whose radius is *Nv* tan *e*. Further, *MSM*_i_(**x**) is the magnitude of the filter response of the Sobel filter at the position (**x**) and *i* (0∼3) because we use four directional Sobel masks (0°, 45°, 90°, 135°). *TMSM*_i_(**x**) is the total magnitude of the filter response of the Sobel filter at the position (**x**); we find the correct gaze position of the user as **xcf***_λ_*,*_θ_* using Equations (3) and (4) within the circle region. Accordingly, we propose Equation (2) using **xcf***_λ_*,*_θ_* instead of **xf***_λ_*,*_θ_* in Equation (1); Figure 2b shows the foveation-based contrast sensitivity mask by our method.

To apply the weighting mask of Figure 2b, the original image that is used to measure eyestrain is decomposed using the four-level DWT based on the Daubechies wavelets, and it is multiplied by the weighting mask of Figure 2b. Finally, the image (spatial domain) applied by the foveation-based contrast sensitivity mask is obtained by the inverse procedure of DWT [11,24].

### 2.4. Measuring Eye BR

In previous research [25], an increase in the BR can be observed as a function of time on task. In other research, Kaneko *et al.* show that blinking frequency increases during prolonged work on visual display terminals [26]. Therefore, we quantitatively measure the degree of a user's eyestrain based on the eye BR in this research. That is, we regard the increase of the BR as an increase in eyestrain in this research.

Using the captured eye image shown in Figure 1, we calculate the BR based on the number of black pixels in the pupil area [9–11]. If the number of black pixels is smaller or larger than the predetermined threshold, the user's eye is determined to be closed or open, respectively. Finally, the BR is calculated by the number of changes in the eye status (close to open) in a time window of 60 s. The status of open to close is not counted. This window is moved by an overlap of 50 s [11]. In previous research, the average BR is usually measured during one min [25]. Therefore, we measured BR per min. In order to remove the effect of the starting position in the time window and analyze the result in detail, we use the scheme of overlapping by 50 s.

## 3. Factors Causing Eyestrain from Viewing 3D Stereoscopic Displays

The image quality of conventional 3D stereoscopic displays can be influenced by various factors, such as camera configurations, image compression, and display [1]. The factors that cause eyestrain in 3D stereoscopic displays can be categorized into three types: spatial distortions, imperfect filters, and stereoscopic disparities [27]. The spatial distortions indicate that the differences in the geometries of the left and right images are caused by various factors, such as image shift, magnification, rotation, blurring, and keystone effect. The imperfect filters represent the photometric asymmetries of the left and right images caused by various factors, such as image brightness, color, contrast, and crosstalk effect. The third category is caused by the inappropriate spatial disparities of the left and right images [27].

Because it has been reported that various factors exist that can damage the 3D perception and cause user eyestrain, it is extremely difficult to consider all causes. Among such causes, the excessive SD is regarded as one of the major factors on eyestrain. However, we use commercial 3D video for experiments, and the level of SD in each image is usually adjusted by post-processing such as to provide eye comfort to the audience for the commercial 3D video. However, in most cases, the CSD in successive images cannot be adjusted because the CSD is also determined by scene changes based on the plot of the video. Therefore, in this study, we measured the change in eyestrain based on the following three 3D factors: degree of CSD, degree of SD, and degree of FCE. The FCE is one of the typical factors that belong to the first category of spatial distortions. As the third category, the excessive SD is regarded as one of the major factors on eyestrain. Additionally, the degree of EC is calculated as a 2D factor to measure the change in eyestrain according to the edge degree both in left and right images of 3D display. Detailed explanations for obtaining ECs are provided in Section 3.3.

### 3.1. Change of Stereoscopic Disparity

SD represents the positional difference between the left and right images in a 3D stereoscopic display, and it affects the user's depth perception. However, an excessive CSD of images can result in eyestrain. Therefore, we measured the relationship between eye BR and the CSD in the image region, as defined by the foveation model shown in Figure 3e.

In the stereoscopic (red-green) image of Figure 3a, the left (green) and right (red) images are separated as shown in Figure 3b. Figure 3b shows the original left and right images, including the foveation point as a red crosshair, respectively. The left image is input into the left eye through the glasses of the green cellophane shown in Figure 1. The right image is input into the right eye through the glasses of the red cellophane shown in Figure 1.

If the gray value in the left image is different from that in the right image even with the same object, these differences in gray level can cause an incorrect disparity calculation. Therefore, brightness normalization is performed on the left and right images by adjusting the mean gray values of the left and right images to be the same, which can reduce the disparity caused by the gray level differences of corresponding pixels in the left and right images. Then, SD is calculated using the stereo matching algorithm from the open source computer vision (OpenCV) library [21], as shown in Figure 3c, which is a modified algorithm of the previous work [28]. Then, the foveation-based contrast sensitivity mask, which is based on the foveation point as a red crosshair shown in Figure 3b and on the edge strength within the circle area of gaze estimation error, shown in Figure 2b is multiplied by the disparity image of Figure 3c in the wavelet domain. Then, the resulting image is transformed into that in the spatial domain. Figure 3d,e shows the disparity images in the spatial domain using the previous foveation method [11] and the proposed method, respectively. Finally, the sum of differences of the corresponding pixel values between the previous disparity image and the current one, where our foveation model is applied, is calculated as the degree of CSD in this research. In detail, we used the successive images of commercial video for experiments. The previous and current disparity images are those at the previous time (t – Δt) and the current time (t), respectively. Further, the degree of SD is calculated by the sum of pixel values in a disparity image where our foveation model is applied. The calculated SD of Figure 3d,e is 3,175,583 and 2,703,869, respectively.

### 3.2. Frame Cancellation Effect

**Figure 4.**
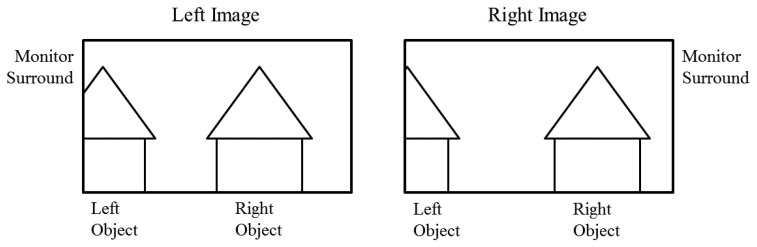
Example of FCE.

In conventional 3D stereoscopic displays, the difference of occlusion of an object in left and right images can occur specifically in the left or right boundary areas of the display. Figure 4 illustrates an example in which more parts of the left object are not shown in the right image because of limitations of the display area. This phenomenon can be another factor that causes eyestrain when viewing a 3D stereoscopic display, because of the differences in the same object between the left and right images. This is called the FCE, and it is known for causing uncomfortable viewing and eyestrain [29].

We compute the FCE as (horizontal resolution of monitor)/2—the gaze position of the X-axis. If the FCE is negative number, we change it to positive by obtaining the absolute value of the FCE. For example, in the case of gaze positions (20, 10) and (1270, 30), the revised FCEs are 620 (1280/2–20) and 630 (1270–1280/2), respectively.

### 3.3. Edge Component

**Figure 5.**
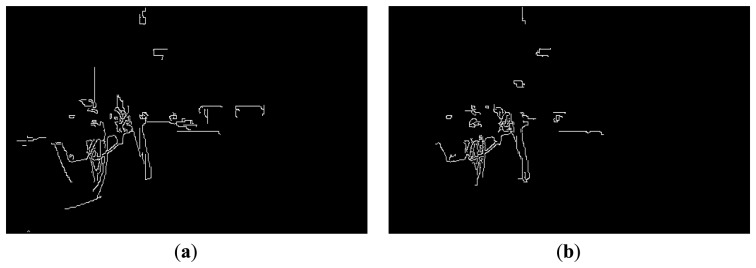
The two edge images obtained from the transformed images (whose contrasts are changed according to our foveation model) using the left and right images from Figure 3b: (**a**) left image; (**b**) right image.

In previous research [11], the relationship between eyestrain and EC was investigated in 2D displays. As indicated in that research, an increase in EC induces a reduction in eyestrain. In our research, we measured the effects of EC on eyestrain in 3D displays, and compared the effect amount to those of other factors, such as CSD, SD, and FCE. The two transformed images, whose contrasts are changed according to our foveation model, are obtained using the left and right images as shown in Figure 3b, and the sum of the edge magnitude calculated with the Canny edge detector is calculated in each image based on the previous research [11]. Finally, these two sums are added, and the total is determined as the degree of EC. The two edge images obtained from the transformed images where our foveation model is applied using the left and right images from Figure 3b are shown in Figure 5a,b, respectively.

In our research, the BR is measured as a sum in the time window of 60 s; this time window is moved by an overlap of 50 s. Therefore, all factors, including CSD, SD, FCE, and EC are measured as a sum in the same time window of 60 s, respectively; this time window is moved by an overlap of 50 s such that we can obtain the value of each factor synchronized with the BR. For example, in the images of 80 s, we can obtain three pairs ((80 − 60)/10 + 1) of the values (BR, CSD), (BR, SD), (BR, FCE), and (BR, EC), respectively. Because the total video length used in our experiment is 25 min 30 s (1530 s), the number of pairs of values ((BR, CSD), (BR, SD), (BR, FCE), and (BR, EC)) is 148 ((1530 − 60)/10 + 1), respectively. With these pairs, we measure correlations similar to Table 1.

In addition, our system does not adapt the video material in real time. Successive images of a user's eye are acquired by synchronizing with the 3D video images in real time. Then, the gaze position, BR, and the corresponding values of CSD, SD, FCE, and EC are obtained through an off-line experiment.

## 4. Experimental Results

The participants in this experiment wore the proposed glasses-type device with red-green glasses shown in Figure 1 to view the test 3D video. Experiments were conducted at the two Z distances between the monitor and the participant at 60 cm and 90 cm, respectively [10,30]. The environmental lighting in the test room was kept constant. In addition, we maintained the room such that it was free of noise, vibration, and bad odors. Six males and six females participated in the test of viewing the 3D display [10,30]. Among them, we analyzed the data from eight participants, excluding the error data from four participants, which were caused by the incorrect detections of pupil and SR regions, or by gazing at regions different from the monitor during the experiments. The participants' mean age was 26.88 years, with a standard deviation of 1.96. We used a commercial 3D movie video for the experiment [10,30].

**Figure 6.**
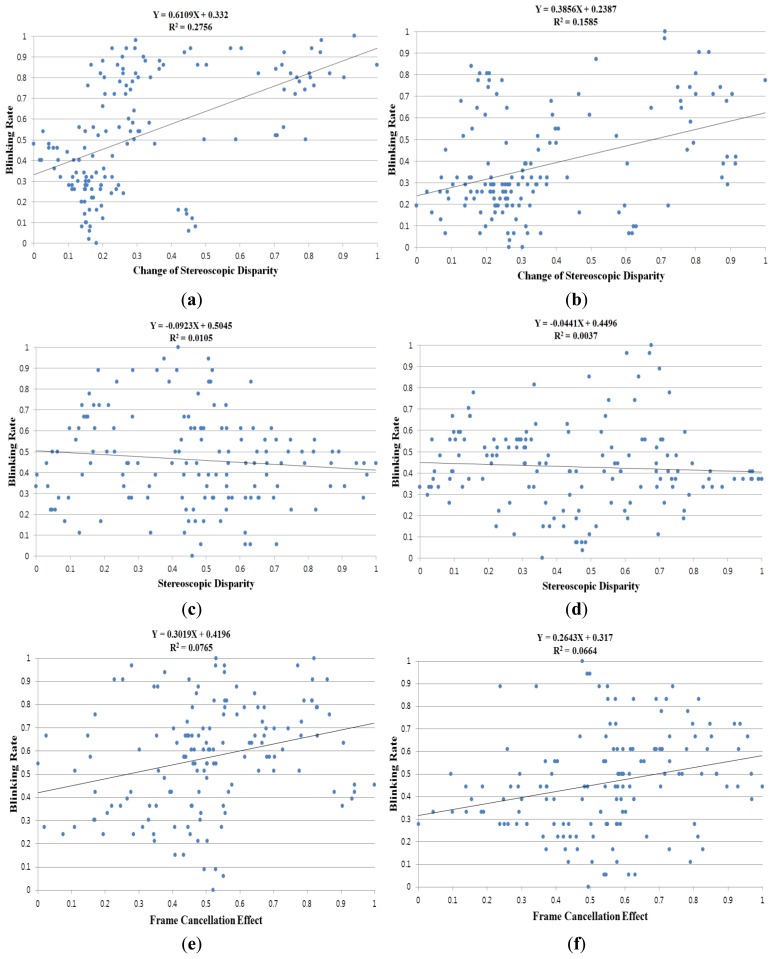

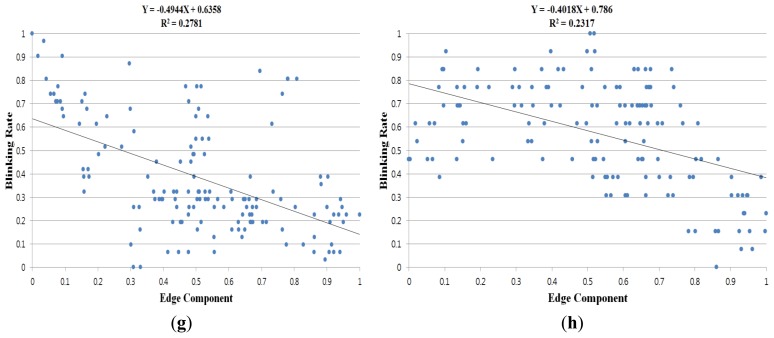
Linear regression results: regression lines showing the correlation (**a**,**b**) between BR and CSD; (**c**,**d**) between BR and SD; (**e**,**f**) between BR and FCE; (**g**,**h**) between BR and EC.

Based on the relationship observed between the eye BR with the function of time on task and the prolonged works on visual display terminals [25,26], we measured the degree of each user's eyestrain according to the three 3D factors (CSD, SD, and FCE) and one 2D factor (EC) as listed in Table 1 and shown in Figure 6. The BR was measured in the time window of 60 s; this time window was moved by an overlap of 50 s. Then, all factors were measured in the time window of 60 s; this time window was moved by an overlap of 50 s. Because there were variations in each factor for each user in terms of BR and other factors, we normalized the values from zero to one based on minimum-maximum scaling. Table 1 represents the results from all the participants.

As listed in Table 1, the average correlation coefficients between BR and factors (CSD, SD, FCE, and EC) by our method were calculated at 0.2841, −0.0299, 0.2214, and −0.2018, respectively. The correlation coefficients close to +1 and −1 represent the cases that are positively and negatively related, respectively. Therefore, an increase in the CSD and the FCE causes an increase in eyestrain (positively related). Here, the increase in the FCE represents the decrease of the horizontal distance between the gaze position and the closer monitor boundary, as explained in Section 3.2. Table 1 demonstrates that the decrease in the EC induces an increase of eyestrain (negatively related), as explained in previous research [11]. The SD is almost uncorrelated to the eyestrain, as demonstrated in Table 1.

In previous research [31], Cho *et al.* measured the relationship between eyestrain and the increase of SD on a sample video by deliberately increasing the level of SD of 3D contents. However, the goal of our research is to measure eyestrain by various factors on conventional 3D video, which is used in the real world, instead of the experimental sample video. Consequently, we used the commercial 3D video for experiments and the level of SD was usually adjusted by post-processing such as to provide eye comfort to the audience for the commercial 3D video. Thus, the experimental results showed that the SD is not related to eyestrain in commercial 3D video in our experiments. In addition, the consequent level of eyestrain in our experiment was low, which reduces the R^2^ value. And, we measured the short-term (almost instant) change of eye BR according to the change of CSD, SD, FCE, and EC during the short time of 1 min, respectively, whereas the long-term change of eye BR was usually measured during the long time in previous research [31]. Therefore, the relationship between the eyestrain based on the BR and the factors is inevitably weak in our experiment, which reduces the R^2^ value, consequently.

The average gradient values for the three 3D factors and the single 2D factor by our method are 0.3084, −0.0506, 0.2440, and −0.1948, respectively. These gradient values were calculated from the fitted line (regression line) on the factor data and the BR, as shown in Figure 6. These results confirm that the CSD have a considerable effect on eyestrain than other factors. In Table 1 and Figure 6, R^2^ indicates the confidence that we have in predictions using the regression lines. If the data is better (more reliably) fitted by the regression, the consequent R^2^ value increases [30]. We obtained 0.1069, 0.0499, 0.0690, and 0.1253 as the average R^2^ values for the 3D factors and the 2D factor by our method, respectively.

In Table 1, we compared the results for the case of using the entire image, the resulting image by previous foveation model [11], and the proposed method. As demonstrated in Table 1, the average correlation coefficient, gradient, and the R^2^ of CSD by our method are larger than those by a previous method [11] and in the case using the entire image without the foveation model. In all the cases by our method, the previous method [11] and using the entire image without the foveation model, the degree of correlation between the eyestrain and the CSD is highest. Those of the FCE, EC, and SD are the second, third, and fourth highest correlations, respectively. Because the FCE value is calculated using only the gaze position without applying the foveation model as shown in Section 3.2, the average correlation coefficient, average gradient, and average R^2^ of the FCE values by our method are same to those by a previous method [11] and those using the entire image.

Figure 6a,b is the results from different participants. Other image pairs (Figure 6c–h) are also those from different users, respectively. Figure 6a,b shows the BR according to the CSD. Figure 6c,d shows the BR according to the SD. Figure 6e,f shows the BR according to the FCE. In Figure 6g,h, the BR is shown according to the EC. From the experimental results, we can confirm that the CSD can affect eyestrain more than other factors, and that eyestrain can be reduced by reducing the CSD and FCE, and by increasing the EC. Given that the proposed method and device can be used with conventional 3D glasses, it is possible to adaptively control the CSD, EC, and FCE based on the BR of the viewer of a 3D stereoscopic display, and thus reduce eyestrain on a real-time basis. Because the FCE is usually caused by the conflict of SD and the occlusion by the monitor boundary [29], if the eyestrain is perceived by our system and the user's gaze position is close to the monitor boundary, the system can lessen the eyestrain by reducing the SD in this case.

For the next analysis, we perform the method of 2^k^ factorial design that is used for measuring the effect of each factor and the interaction effect on the system performance [10,32]. Because the factors of the largest and smallest effect on the eyestrain are CSD and SD, respectively, as summarized in Table 1, we perform the method of 2^k^ factorial design with these two factors. First, we measure the maximum and minimum SD from the test images. Based on the medium value of the maximum and minimum SDs, we divide the images into two types, Large and Small, as listed in Table 2. Using the same method, the images are also classified into the types Large and Small based on CSD, as listed in Table 2. Consequently, we can obtain four cases of image categories as (Large, Large), (Small, Large), (Large, Small), and (Small, Small), respectively, based on SD and CSD. In each case, we measure the average BR; the four BRs are listed in Table 2.

Then, the BR (*y*) can be regressed based on the nonlinear regression model in Equation (5):

(5) $$y=q_{0}+q_{A}x_{A}+q_{B}x_{B}+q_{AB}x_{A}x_{B}$$

To perform the method of 2^k^ factorial design, the *x_A_* and *x_B_* are defined as (−1, −1), (1, −1), (−1, 1), and (1, 1) in the cases of (Large, Large), (Small, Large), (Large, Small), and (Small, Small), respectively. From them, Equations (6) and (7) are obtained:

(6) $$\begin{array}{c} y_{1}=q_{0}-q_{A}-q_{B}+q_{AB}\\ y_{2}=q_{0}+q_{A}-q_{B}-q_{AB}\\ y_{3}=q_{0}-q_{A}+q_{B}-q_{AB}\\ y\_4=q\_0+q\_A+q\_B+q\_AB \end{array}$$

(7) $$\left\lceil \begin{array}{c} \begin{array}{cccc} 1 & -1 & -1 & 1 \end{array}\\ \begin{array}{cccc} 1 & 1 & -1 & -1 \end{array}\\ \begin{array}{cccc} 1 & -1 & 1 & -1 \end{array} \end{array}\right]\;\left[\begin{array}{c} q_{0}\\ q_{A}\\ q_{B}\\ q_{AB} \end{array}\right]=\left[\begin{array}{c} y_{1}\\ y_{2}\\ y_{3}\\ y_{4} \end{array}\right]$$

In Equation (7)*y*_1_–*y*_4_ are 0.4842, 0.5871, 0.4703, and 0.4098, respectively, based on Table 2. Based on *y*_1_–*y*_4_ and Equation (7), the *q*_0_, *q_A_*, *q_B_*, and *q_AB_* are calculated as 0.4878, 0.0106, −0.0478, and −0.0410, respectively. The *q*_0_, *q_A_*, *q_B_*, and *q_AB_* represent the average value of *y*_1_–*y*_4_, the effect of SD, the effect of CSD, and the interaction effect of SD and CSD, respectively. The sum of squares total (*SST*) is obtained to calculate the proportion of the effect for each factor [10,32]:

(8) $$\text{SST}=2^{2}q_{A}^{2}+2^{2}q_{B}^{2}+2^{2}q_{AB}^{2}=\text{SSA}+\text{SSB}+\text{SSAB}$$

Using Equation (8), the sum of squared factor A (*SSA*), sum of squared factor B (*SSB*), and sum of squared factor AB (*SSAB*) are 0.0004, 0.0091, and 0.0067, respectively. The effect ratios of each factor are calculated by 100 × *SSA*/*SST* (%), 100 × *SSB*/*SST* (%), and 100 × *SSAB*/*SST* (%), respectively. Consequently, they are 2.76%, 56.19%, and 41.04%, respectively. Based on these values, we can reach the following conclusions:

- The effect of factor B (CSD) is approximately 20.4 (56.19/2.76) times greater than that of factor A (SD).
- The effect of factor B (CSD) is approximately 1.4 (56.19/41.04) times greater than that of the interaction effect of factors A and B.
- The interaction effect of factors A and B is approximately 14.9 (41.04/2.76) times greater than that of factor A (SD).

From these conclusions, we can confirm that the CSD has more of an effect on eyestrain than the SD in terms of the method of 2^k^ factorial design. In addition, we provide descriptive statistics based on effect size [33]. In statistics, an effect size is used as a measure to demonstrate the strength of a given phenomenon, and the effect size calculated from data is regarded as a descriptive statistic [33]. Based on [33], we show that the effect size based on Cohen's *d* is calculated as the difference value between two means divided by a standard deviation for the data. Based on the previous research [34], we define the values of 0.2, 0.5, and 0.8 for Cohen's *d* value as small, medium, and large, respectively. In [33], for Cohen's *d*, an effect size of 0.2 to 0.3 might be a small effect. A value of around 0.5 might be a medium effect, and that of 0.8 to infinity (the *d* might be larger than one) might be a large effect.

As listed in Table 3, we include the measured Cohen's *d* between two factors that cause eyestrain with the results of our method from Table 1. For example, Cohen's *d* between the average correlation coefficient of CSD and that of SD is 1.5145, which is closer to 0.8 (large effect) than to 0.2 (small effect) or to 0.5 (medium effect). Therefore, we can confirm that there exists a difference between the average correlation coefficient of CSD and that of SD as a large effect size. Another example is that Cohen's *d* between the average gradient of CSD and that of FCE is 0.3345, which closer to 0.2 than to 0.5 or to 0.8. Thus, we can confirm that there exists a difference between the average gradient of CSD and that of FCE as a small effect size.

Here, the average correlation coefficient and the average gradient usually show a degree of correlation between the eyestrain measured using the eye BR and the factor. By referring to the results of Tables 1 and 3, we can confirm that the degree of correlation between the eyestrain and the CSD is highest. Those of the FCE, EC, and SD are the second, third, and fourth highest correlations, respectively. In addition, the difference between the degrees of correlation of two factors is shown as the effect size of Table 3. That is, there exists a difference between the degree of correlation of CSD and that of SD as a large effect size. In addition, there exists a difference between the degree of correlation of CSD and that of EC (or FCE) as a small or medium effect size.

For comparison, we measured the relationship between the BR and various factors through additional experiments with the experimental sample video. The name of sample video is *Summer in Heidelberg* and we obtained permission from the video copyright owner [35].

**Figure 7.**
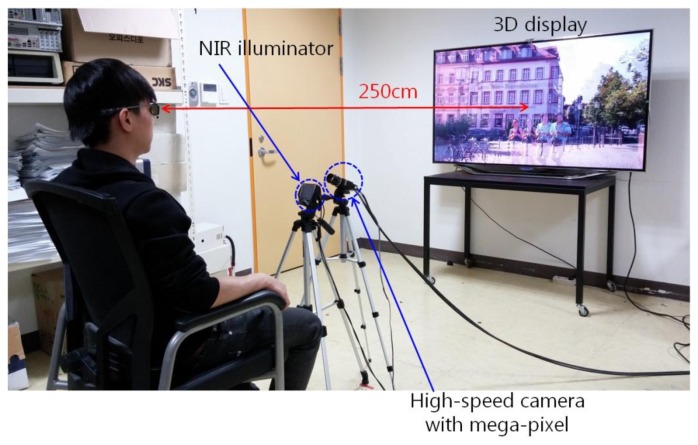
Example of experimental setup for measuring eyestrain on 3D display using a remote (lab-made) gaze tracking system.

A total of fourteen people participated in the experiment, and each person watched the 3D video using active shutter glasses for 30 min. The user's eye images were captured at a speed of about 70 fps using a remote (lab-made) gaze tracking system as shown in Figure 7. The Z distance between the user and the display is approximately 250 cm. For the experiment, we used a commercial 3D TV display of 60 inches with an image resolution of 1920 × 1080 pixels and a refresh rate of 48 Hz (24 Hz for the left image and 24 Hz for the right image, respectively).

As listed in Table 4, we can confirm that the correlation coefficient value of SD is higher than those of other factors with the experimental 3D sample video (where the level of SD of 3D contents is deliberately increased) while that of CSD is higher than those of other factors with the commercial 3D video as shown in Table 1.

In Table 5, we include the measured Cohen's *d* between two factors that cause eyestrain with the results of our method as listed in Table 4. By referring to the results of Tables 4 and 5, we can confirm that the degree of correlation between the eyestrain and the SD is highest. Those of the CSD, EC, and FCE are the second, third, and fourth highest correlations, respectively. In addition, the difference between the degrees of correlation of two factors is shown as the effect size of Table 5. That is, there exists a difference between the degree of correlation of SD and that of other factors (CSD, EC, and FCE) as a medium effect size. The differences between the degrees of correlation of other two factors (CSD, EC, and FCE) are shown as small effect size.

**Figure 8.**
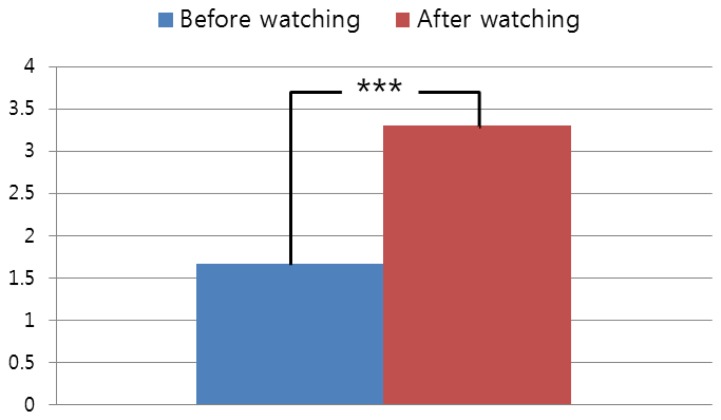
Result of subjective evaluation with the commercial 3D video of Table 1. (***: significant at a confidence level of *p* < 0.01).

For the next experiment, we include the subjective evaluations of our system as shown in Figure 8 with a two-tailed T-test.

For the subjective evaluation, the following six questions were answered using a 10-point scale, where one corresponds to *not at all*, and ten corresponds to *yes*, *very much*) [10]; these questions were designed based on previous research [36].

1. I have difficulties seeing.
2. I have a strange feeling around the eyes.
3. My eyes feel tired.
4. I feel numb.
5. I feel dizzy looking at the screen.
6. I have a headache.

Subjective tests were performed before and after watching the 3D video. All the relationships between BR and the 3D/2D factors from Table 1 were measured from the eye images captured while the users watched the 3D video. Further, these 3D/2D factors could not be controlled because the commercial 3D movie was used for our experiment. Therefore, we performed the subjective tests before and after watching the 3D video.

**Figure 9.**
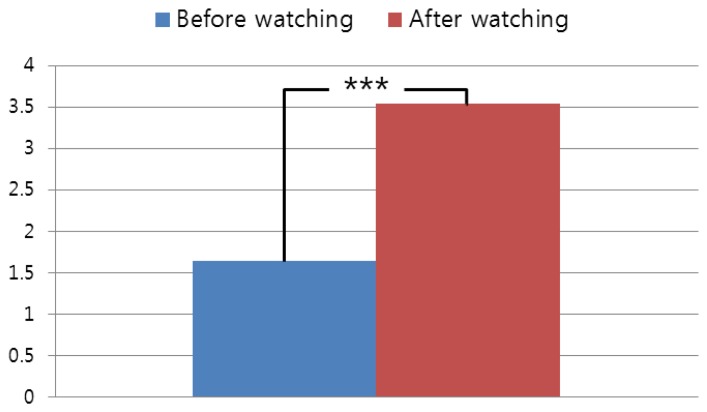
Result of subjective evaluation of Figure 7. (***: significant at a confidence level of *p* < 0.01).

As shown in Figure 8, the subjective eyestrain after watching the 3D video is higher than that before watching the 3D video. Because the *p*-value is 0.0091 and it is less than 0.01, we can confirm that the subjective eyestrain after watching the 3D video is significantly higher than before watching the 3D video, with a confidence level of 99% (0.01) [37]. We formed the null-hypothesis that there is no difference between the subjective eyestrain before and after watching the 3D video. According to the principle of T-test [37], if the *p*-value is less than the confidence level, the null-hypothesis is rejected, which indicates that there exists a difference between the subjective eyestrain before and after watching the 3D video. In addition, we include the subjective evaluations before and after watching the 3D video of Figure 7 as shown in Figure 9.

As shown in Figure 9, the subjective eyestrain after watching the 3D video is higher than that before watching the 3D video. Because the *p*-value is 0.0001 and it is less than 0.01, we can confirm that the subjective eyestrain after watching the 3D video is significantly higher than before watching the 3D video, with a confidence level of 99% (0.01) [37].

In our experiment, the successive images of the user's eye are acquired by synchronizing with the 3D video images at a speed of 15 fps. From that, 15 gaze points are obtained per 1 s. We regard the position calculated based on pupil center and four SRs at every eye frame as the user's gaze point. Here, we do not consider eye jittering during fixations because of drift, tremor and involuntary micro-saccades [38], and saccadic movement in our experiment. Because the gaze position is measured on every eye image, and the eye image is synchronized with the 3D video image, we can relate the gaze point of the eye image to that in the 3D video image.

In order to consider the eye jittering and saccadic movement, eye images that are captured at faster speed are necessary. For that, a high-speed camera is required for the eye capturing device, which increases the size and weight of the eye capturing device shown in Figure 1. Wearing this type of heavy device when watching a display for an extended time can inevitably increase a user's discomfort, which can cause an inaccurate measurement of the user's eyestrain. Therefore, we use a small and light-weighted web-camera, through which it is difficult to consider the eye jittering and saccadic movement because of the low capturing speed of 15 fps.

With the eye images of 15 fps (Table 1), we reduce the saccade movement on the eye gaze point by using the revised method of previous research [38], by which a more accurate gaze point can be obtained. According to this compensated gaze point, we obtain the relationship between the BRs and various factors in Table 6. As listed in Table 6, the results based on the gaze position that is compensated are similar to those of Table 1 based on the uncompensated gaze position. Because the FCE value is calculated using only the gaze position without applying the foveation model as shown in Section 3.2, the average correlation coefficient, average gradient, and average R^2^ of FCE values by our method are same to those by a previous method [11] and those using the entire image.

## 5. Discussions

**Figure 10.**
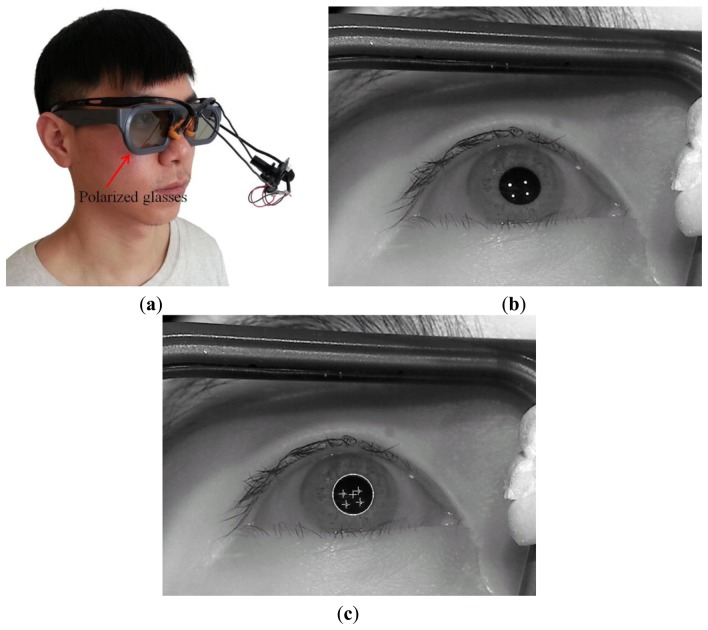
Examples of applying our device and method to the polarized glasses for 3D stereoscopic display: (**a**) the case of using our device with polarized glasses for 3D stereoscopic display; (**b**) the captured eye image; (**c**) the resulting image from successfully detecting the pupil center with four SR positions.

**Figure 11.**
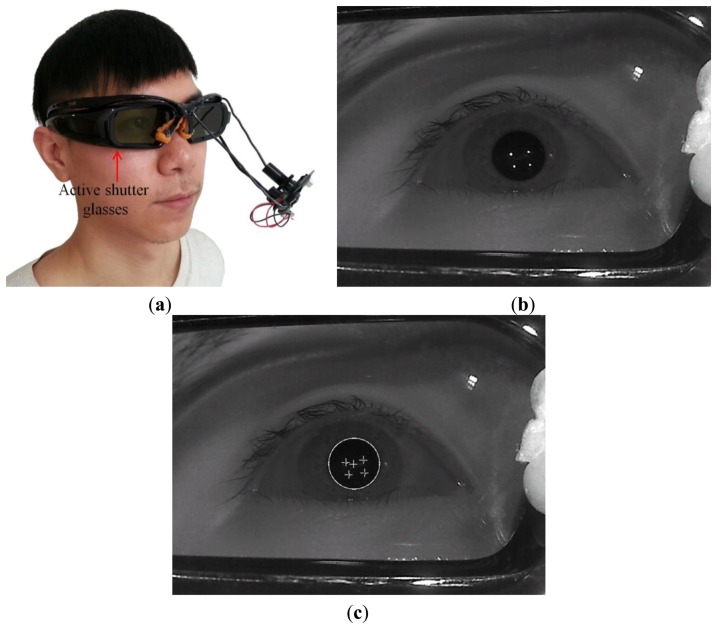
Examples of applying our device and method to the active shutter glasses for 3D stereoscopic display: (**a**) the case of using our device with active shutter glasses for 3D stereoscopic display; (**b**) the captured eye image; (**c**) the resulting image from successfully detecting the pupil center with four SR positions.

Our eye capturing device and method can be applied easily to other stereoscopic viewing modalities. Figure 10a shows the case of using our device with polarized glasses for 3D stereoscopic display. Figure 10b,c shows the captured eye image and the resulting image from successfully detecting the pupil center with four SR positions, respectively. In addition, Figure 11a shows the case of using our device with active shutter glasses for 3D stereoscopic display. Figure 11b,c shows the captured eye image and the resulting image from successfully detecting the pupil center with four SR positions, respectively. From these images, we can confirm that our eye capturing device and method can be easily applied to other stereoscopic viewing modalities.

Because the implementation algorithm and code of [29] are not freely available, it is difficult to measure the performance by combining the method of [29] and our system. In addition, they proposed a novel method (stereo compatible volume clipping (SCVC)) to avoid frame cancellation by rendering only the part of the viewing volume (SCV) that is free of conflict using the clipping methods available in standard real-time 3D application programming interfaces (APIs) [29]. That is, Ardouin *et al.*'s method can be used for 3D graphic content such as virtual reality and game content whose rendering volume can be controlled by their algorithm. However, our system is used for actual video images captured through a camera where it is difficult to control rendering the part of the viewing volume.

In our research, the proposed system does not adapt the video material in real time. The successive images of the user's eye are acquired by synchronizing with the 3D video images in real time. Then, the gaze position, BR, and the corresponding values of CSD, SD, FCE, and EC are obtained through an off-line experiment.

In future work, our system can be used as a real-time system that can control various factors (CSD, SD, FCE, and EC) according to the user's eyestrain as follows. If the eyestrain is perceived by our system based on the increased number of BR, our system can send the signal to 3D TV where the CSD should be reduced. Because the FCE is usually caused by the conflict of SD and the occlusion by the monitor boundary [29], if the eyestrain is perceived by our system and the user's gaze position is close to the monitor boundary, our system can send the signal to the 3D TV. Then, the 3D TV can lessen the eyestrain by reducing the SD in this case.

Because the BR and all the factors (CSD, SD, FCE, and EC) were measured in the time window of 60 s and this time window was moved by an overlap of 50 s, our system can send the eyestrain signal and request adaptation of the 3D video to the 3D TV system every 10 s when used in a real-time system.

The relationships between the factors (CSD, SD, FCE, and EC) and eyestrain measured by BR are calculated with the data which are obtained while each user is watching the 3D video. Because the subjective test cannot be performed while watching the 3D video (it can be done only before or after watching video), these relationships cannot be measured by the subjective test. Therefore, we performed the subjective test for measuring eyestrain before and after watching the 3D video. From the Figures 8 and 9, we can confirm that the subjective eyestrain after watching the 3D video is higher than that before watching the 3D video with a confidence level of 99% (0.01). This is another conclusion of our research.

Therefore, the first conclusion of our research is that with the commercial 3D video, the degree of correlation between the eyestrain and the CSD is highest. Those of the FCE, EC, and SD are the second, third, and fourth highest correlations, respectively. That is because the level of SD in each image is usually adjusted by post-processing such as to provide eye comfort to the audience for the commercial 3D video. However, in most cases, the CSD in successive images cannot be adjusted because the CSD is also determined by scene changes based on the plot of the video. The second conclusion is that with the experimental 3D sample video (where the level of SD of 3D contents is deliberately increased), the degree of correlation between the eyestrain and the SD is highest. Those of the CSD, EC, and FCE are the second, third, and fourth highest correlations, respectively. The third conclusion is that the subjective eyestrain after watching the 3D video (in both cases of commercial and experimental 3D sample videos) is higher than that before watching the 3D video with a confidence level of 99% (0.01).

Lee *et al.* applied an eye foveation model that considers the user's gaze position and gaze error when measuring the eyestrain according to variance of hue, edge, and motion information in 2D displays [11]. Although they define the circle region where the user's gaze position exists by considering the gaze estimation error, they do not determine the gaze position that has a higher probability of being the correct one inside the circle region. However, we find the gaze position that has a higher probability of being the correct one where the magnitude of the filter response by four directional Sobel masks is maximized within the circle region as shown in Equations (2)–(4). Therefore, the contrast sensitivity mask in wavelet domain by our method of Figure 2b is different from that by previous method [11] of Figure 2a. By comparing the correlations between the BR and factors by our method to those by previous method [11] as shown in Tables 1 and 6, we can confirm that the correlation by our method is higher than that by the previous one [11]. These are the main differences, improvements and contributions of our research compared to previous one [11].

Other differences, improvements and contributions of our research compared to previous one [11] are as follows. In previous research [11], they do not measure the eyestrain according to various factors in the 3D stereoscopic display, but evaluate the eyestrain according to 2D factors such as variance of hue, edge and motion information in 2D display. Therefore, they obtained the following conclusion. The decrement of the variance of hue value in a 2D display induces a decrease in eyestrain. In addition, increased edge and motion components induce a reduction in eyestrain in as 2D display.

However, we evaluate the correlation between the degree of eyestrain and the causal factors of visual fatigue, such as the degree of CSD, SD, FCE, and EC in the 3D stereoscopic display. In addition, by comparing the eyestrain in conventional 3D video and experimental 3D sample video, we analyze the characteristics of eyestrain according to various factors and types of 3D video. And, by comparing the eyestrain with or without the compensation of eye saccades movement in 3D video, we analyze the characteristics of eyestrain according to the types of eye movements in 3D video. In addition, we show the applicability of our method to the 3D system based on the polarized glasses and active shutter glasses as shown in Figures 10 and 11. Therefore, we obtained the above three conclusions different from that of previous work [11].

The last difference, improvement and contribution of our research compared to previous one [11] are as follows. In previous research [11], they do not perform the 2^k^ factorial design, descriptive statistics based on effect size, and subjective evaluation with T-test. However, we did them as shown in Tables 2, 3 and 5 and Figures 8 and 9 in order to enhance the credibility of the measured results by our method. Therefore, we confirm that our research is different from [11], and we made the improvements and contributions over the previous work [11].

In previous research [15], Bang *et al.* presented a new computer interface that combines gaze tracking with brainwave measurements in an integrated head-mounted device for the navigation in 3D world. We just adopt the method of attaching four NIR illuminators at the four corners of monitor, locating the four corneal SRs in the eye image, and Kappa calibration from [15] as shown in Section 2.2. Their research is not about the eyestrain measurement in 2D or 3D display [15], and it is completely different from our research.

## 6. Conclusions

In this paper, we proposed a new method for measuring the degree of eyestrain caused by viewing 3D stereoscopic display based on eye BR, considering both the viewer's gaze position and edge information. To quantify the eyestrain on the viewer of a 3D display, we used a pupil detection device that consists of eyeglasses and a web camera. We measured the viewer's BR to determine the extent of eyestrain. Then, we evaluated the correlation between the extent of eyestrain and the three 3D causal factors (CSD, SD, and FCE) and one 2D factor (EC) of 3D display. Our experimental results showed that the CSD has a significantly larger effect on eyestrain than the other factors. In addition, by comparing the eyestrain in conventional 3D video and experimental 3D sample video, we analyzed the characteristics of eyestrain according to various factors and the types of 3D video. Further, by comparing the eyestrain with or without the compensation of eye saccades movement in 3D video, we analyzed the characteristics of eyestrain according to the types of eye movements in 3D video.

Although we measured the eyestrain using the foveation model considering the fovea centralis region, the peripheral vision can also be a factor that causes eyestrain in the case of motion and in real-time applications, if not controlled. Therefore, this is the limitation of our research and we would leave this issue open what would happen if a global model is used instead of the strain in the “fovea centralis” region. For future work, we would conduct research to determine whether our system can be used as a real-time system that can control various factors (CSD, SD, FCE, and EC), according to user's eyestrain. In addition, we plan to combine the measurements of eye BR with brainwaves or other physiological signals for a more accurate evaluation of eyestrain.

## Figures and Tables

**Table 1.** Correlation between BR and factors for causing eyestrain on 3D display.

**Eye Responses**	**3D or 2D Factors**		**Average Correlation Coefficient (Standard Deviation)**	**Average Gradient (Standard Deviation)**	**Average R^2^ (Standard Deviation)**
BR	CSD	By our method	0.2841 (0.1730)	0.3084 (0.2086)	0.1069 (0.1085)
		By previous method [11]	0.2746 (0.1737)	0.2976 (0.2043)	0.1018 (0.1015)
		By using entire image (without foveation model)	0.2734 (0.1752)	0.2963 (0.2065)	0.1016 (0.1020)
	SD	By our method	−0.0299 (0.2368)	−0.0506 (0.2433)	0.0499 (0.0471)
		By previous method [11]	−0.0330 (0.2356)	−0.0547 (0.2423)	0.0497 (0.0455)
		By using entire image (without foveation model)	−0.0326 (0.2351)	−0.0544 (0.2419)	0.0494 (0.0451)
	FCE	By our method	0.2214 (0.1512)	0.2440 (0.1753)	0.0690 (0.0884)
		By previous method [11]			
		By using entire image (without foveation model)			
	EC	By our method	−0.2018 (0.3065)	−0.1948 (0.2828)	0.1253 (0.1283)
		By previous method [11]	−0.1826 (0.2821)	−0.1824 (0.2672)	0.1030 (0.1074)
		By using entire image (without foveation model)	−0.1548 (0.2333)	−0.1441 (0.2110)	0.0716 (0.0909)

**Table 2.** Average BR in terms of CSD and SD.

**CSD**	**SD**	

	Large	Small
Large	0.4842	0.5871
Small	0.4703	0.4098

**Table 3.** The measured Cohen's *d* between two factors that cause eyestrain with the results of Table 1.

**Two Factors**		**Cohen's *d***	**Effect Size**
CSD *vs.* SD	Average Correlation Coefficient	1.5145	Large
	Average Gradient	1.5843	Large

CSD *vs.* FCE	Average Correlation Coefficient	0.3860	Medium
	Average Gradient	0.3345	Small

CSD *vs.* EC	Average Correlation Coefficient	0.3307	Small
	Average Gradient	0.4573	Medium

SD *vs.* FCE	Average Correlation Coefficient	1.2650	Large
	Average Gradient	1.3892	Large

SD *vs.* EC	Average Correlation Coefficient	0.8460	Large
	Average Gradient	0.9303	Large

FCE *vs.* EC	Average Correlation Coefficient	0.0811	Small
	Average Gradient	0.2091	Small

**Table 4.** Correlation between BR and factors for causing eyestrain on 3D display with experimental sample video by our method.

**Eye Responses**	**3D or 2D Factors**	**Average Correlation Coefficient (Standard Deviation)**	**Average Gradient (Standard Deviation)**	**Average R^2^ (Standard Deviation)**
BR	CSD	0.2196 (0.1026)	0.1903 (0.0825)	0.0580 (0.0525)
	SD	0.2584 (0.1182)	0.2536 (0.1165)	0.0798 (0.0677)
	FCE	0.1782 (0.1376)	0.1747 (0.1501)	0.0493 (0.0624)
	EC	−0.1968 (0.1363)	−0.1890 (0.1383)	0.0560 (0.0639)

**Table 5.** The measured Cohen's *d* between two factors causing eyestrain with the results of Table 4.

**Two Factors**		**Cohen's *d***	**Effect Size**
SD *vs.* CSD	Average Correlation Coefficient	0.3511	Medium
	Average Gradient	0.3593	Medium

SD *vs.* EC	Average Correlation Coefficient	0.4834	Medium
	Average Gradient	0.3615	Medium

SD *vs.* FCE	Average Correlation Coefficient	0.6252	Medium
	Average Gradient	0.4673	Medium

CSD *vs.* EC	Average Correlation Coefficient	0.1891	Small
	Average Gradient	0.0348	Small

CSD *vs.* FCE	Average Correlation Coefficient	0.3405	Small
	Average Gradient	0.1501	Small

EC *vs.* FCE	Average Correlation Coefficient	0.1353	Small
	Average Gradient	0.1047	Small

**Table 6.** Correlation between BR and factors for causing eyestrain on 3D display with the commercial 3D video used in Table 1 based on the gaze position that is compensated.

**Eye Responses**	**3D or 2D Factors**		**Average Correlation Coefficient (Standard Deviation)**	**Average Gradient (Standard Deviation)**	**Average R^2^ (Standard Deviation)**
BR	CSD	By our method	0.2796 (0.1692)	0.3032 (0.1963)	0.1032 (0.0981)
		By previous method [11]	0.2780 (0.1719)	0.3016 (0.1994)	0.1031 (0.0991)
		By using whole image (without foveation model)	0.2734 (0.1752)	0.2963 (0.2065)	0.1016 (0.1020)
	SD	By our method	−0.0330 (0.2357)	−0.0545 (0.2422)	0.0497 (0.0454)
		By previous method [11]	−0.0326 (0.2352)	−0.0543 (0.2419)	0.0495 (0.0450)
		By using whole image (without foveation model)	−0.0326 (0.2351)	−0.0544 (0.2419)	0.0494 (0.0451)
	FCE	By our method	0.2226 (0.1543)	0.2470 (0.1796)	0.0704 (0.0898)
		By previous method [11]			
		By using whole image (without foveation model)			
	EC	By our method	−0.2527 (0.3282)	−0.2285 (0.2893)	0.1581 (0.1807)
		By previous method [11]	−0.2424 (0.3319)	−0.2251 (0.3014)	0.1551 (0.1665)
		By using whole image (without foveation model)	−0.1548 (0.2333)	−0.1441 (0.2110)	0.0716 (0.0909)

## References

[1] Meesters L.M.J., IJsselsteijn W.A., Seuntiëns P.J.H. (2004). A survey of perceptual evaluations and requirements of three-dimensional TV. IEEE Trans Circuits Syst Video Technol..

[2] Oyamada H., Iijima A., Tanaka A., Ukai K., Toda H., Sugita N., Yoshizawa M., Bando T. (2007). A pilot study on pupillary and cardiovascular changes induced by stereoscopic video movies. J. Neuroeng Rehabil..

[3] Li H.-C.O., Seo J., Kham K., Lee S. Measurement of 3D Visual Fatigue Using Event-Related Potential (ERP): 3D Oddball Paradigm.

[4] Hagura H., Nakajima M. (2006). Study of asthenopia caused by the viewing of stereoscopic images: Measurement by MEG and other devices. Proc SPIE.

[5] Kim C.J., Park S., Won M.J., Whang M., Lee E.C. (2013). Autonomic nervous system responses can reveal visual fatigue induced by 3D displays. Sensors.

[6] Fukushima T., Torii M., Ukai K., Wolffsohn J.S., Gilmartin B. (2009). The relationship between CA/C ratio and individual differences in dynamic accommodative responses while viewing stereoscopic images. J. Vision.

[7] Shibata T., Kawai T., Ohta K., Otsuki M., Miyake N., Yoshihara Y., Iwasaki T. (2005). Stereoscopic 3-D display with optical correction for the reduction of the discrepancy between accommodation and convergence. J. Soc. Inf. Disp..

[8] Ukai K., Howarth P.A. (2008). Visual fatigue caused by viewing stereoscopic motion images: Background, theories, and observations. Displays.

[9] Lee E.C., Lee S.M., Won C.S., Park K.R. (2009). Minimizing eyestrain on LCD TV based on edge difference and scene change. IEEE Trans. Consum. Electron..

[10] Lee E.C., Heo H., Park K.R. (2010). The comparative measurements of eyestrain caused by 2D and 3D displays. IEEE Trans. Consum. Electron..

[11] Lee W.O., Heo H., Lee E.C., Park K.R. (2013). Minimizing eyestrain on a liquid crystal display considering gaze direction and visual field of view. Opt. Eng..

[12] Van der Linde I. (2004). Multi-resolution image compression using image foveation and simulated depth of field for stereoscopic displays. Proc. SPIE.

[13] Çöltekin A. Space-Variant Image Coding for Stereoscopic Media.

[14] Duchowski A.T., Çöltekin A. (2007). Foveated gaze-contingent displays for peripheral LOD management, 3D visualization, and stereo imaging. ACM Trans. Multimed. Comput. Commun. Appl..

[15] Bang J.W., Lee E.C., Park K.R. (2011). New computer interface combining gaze tracking and brainwave measurements. IEEE Trans. Consum. Electron..

[16] Lee J.W., Cho C.W., Shin K.Y., Lee E.C., Park K.R. (2012). 3D gaze tracking method using purkinje images on eye optical model and pupil. Opt. Lasers Eng..

[17] Geisler W.S., Perry J.S. (1998). A real-time foveated multiresolution system for low-bandwidth video communication. Proc SPIE.

[18] Wang Z., Lu L., Bovik A.C. (2003). Foveation scalable video coding with automatic fixation selection. IEEE Trans. Image Process..

[19] Chang E.-C., Mallat S., Yap C. (2000). Wavelet foveation. Appl. Comput. Harmonic Anal..

[20] Baddeley R.J., Tatler B.W. (2006). High frequency edges (but not contrast) predict where we fixate: A bayesian system identification analysis. Vis. Res..

[21] OpenCV. http://docs.opencv.org/modules/calib3d/doc/camera_calibration_and_3d_reconstruction.html#stereosgbm.

[22] Itti L., Koch C., Niebur E. (1998). A model of saliency-based visual attention for rapid scene analysis. IEEE Trans. Pattern Anal. Mach. Intell..

[23] Itti L., Koch C. (2001). Computational modelling of visual attention. Nat. Rev. Neurosci..

[24] Gonzalez R.C., Woods R.E. (2002). Digital Image Processing.

[25] Stern J.A., Boyer D., Schroeder D. (1994). Blink rate: A possible measure of fatigue. Hum. Factors.

[26] Kaneko K., Sakamoto K. (2001). Spontaneous blinks as a criterion of visual fatigue during prolonged work on visual display terminals. Percept. Mot. Skills.

[27] Kooi F.L., Toet A. (2004). Visual comfort of binocular and 3D displays. Displays.

[28] Hirschmüller H. (2008). Stereo processing by semiglobal matching and mutual information. IEEE Trans. Pattern Anal. Mach. Intell..

[29] Ardouin J., Lécuyer A., Marchal M., Marchand E. Design and Evaluation of Methods to Prevent Frame Cancellation in Real-Time Stereoscopic Rendering.

[30] Lee E.C., Lee J.W., Park K.R. (2011). Experimental investigations of pupil accommodation factors. Invest Ophthalmol. Vis. Sci..

[31] Cho S.-H., Kang H.-B. The measurement of eyestrain caused from diverse binocular disparities, viewing time and display sizes in watching stereoscopic 3D content.

[32] 2^k^ Factorial Designs. http://www.cs.wayne.edu/~hzhang/courses/7290/Lectures/5%20-%202%5Ek%20Factorial%20Designs.pdf.

[33] Effect Size. http://en.wikipedia.org/wiki/Effect_size#Cohen.27s_d.

[34] Cohen J. (1992). A power primer. Psychol. Bull..

[35] Dongleware. http://www.dongleware.de.

[36] Wolfgang J.-K. (1990). On the preferred viewing distances to screen and document at VDU workplaces. Ergonomics.

[37] Moser B.K., Stevens G.R., Watts C.L. (1989). The Two-Sample T Test Versus Satterthwaite's Approximate F test. Commun. Stat..

[38] Kumar M., Klingner J., Puranik R., Winograd T., Paepcke A. Improving the accuracy of gaze input for interaction.

